# Supplementary material for: Endoplasmic Reticulum Stress Cooperates in Silica Nanoparticles-Induced Macrophage Apoptosis via Activation of CHOP-Mediated Apoptotic Signaling Pathway
Source: Int J Mol Sci. 2019 Nov 21;20(23):5846. doi: 10.3390/ijms20235846 (PMC6929173; doi:10.3390/ijms20235846)
Supplement: Supplementary file 1 [file ijms-20-05846-s001.pdf]

Supplementary material

Endoplasmic reticulum stress cooperates in silica nanoparticles-induced macrophage apoptosis via activation of CHOP-mediated apoptotic signaling pathway

Fenglei Chen[1, 2]*, Jiaqi Jin[1, 2], Jiahui Hu[1, 2], Yujing Wang[1, 2], Zhiyu Ma[1, 2] and Jinlong Zhang[1, 2]

[1] College of Veterinary Medicine, Yangzhou University, Yangzhou, 225009, Jiangsu, China; e-mail@e-mail.com

[2] Jiangsu Co-innovation Center for Prevention and Control of Important Animal Infectious Diseases and Zoonoses, Yangzhou, 225009, Jiangsu, China;

* Correspondence: flchen@yzu.edu.cn (F.C.); Tel.: +86-514-8797-9030

1. Results

A

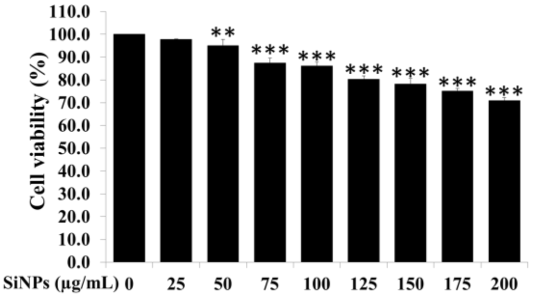

B

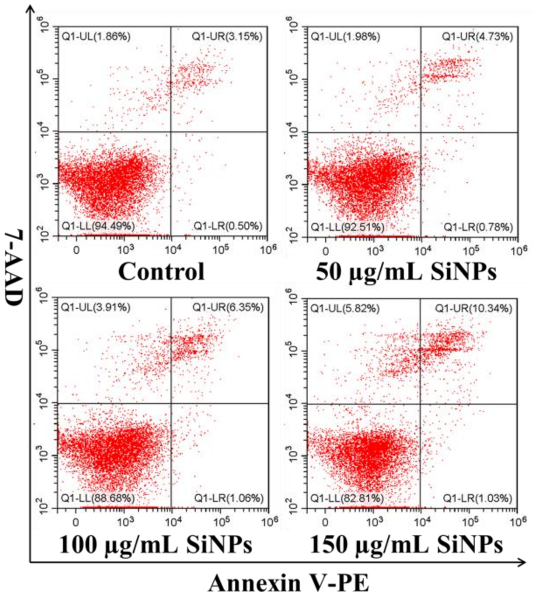

C

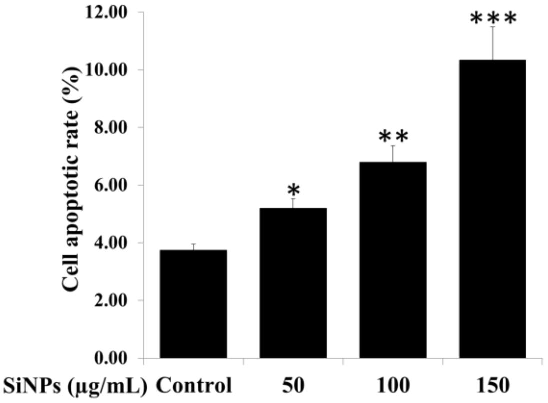

Figure S1. SiNPs decreased cell viability and induced cell apoptosis in RAW 264.7 macrophage cells. (**A**) SiNPs reduced cell viability of RAW 264.7 macrophage cells in a dose-dependent manner. Cells were treated with 0, 25, 50, 75, 100, 125, 150, and 200 μg/mL SiNPs for 12 h and then processed via the CCK-8 assay. (**B, C**) Cell apoptosis was detected via flow cytometry. After exposure to 0, 50, 100, and 150 μg/mL SiNPs for 12 h, RAW 264.7 macrophage cells were collected for Annexin V-PE/7-AAD staining. UL quadrant is the part of cell death caused by mechanical damage or necrotic cells, UR quadrant is the part of late apoptotic cells, LL quadrant is the part of the normal cells, and LR quadrant is the part of early apoptotic cells. The number of cell apoptosis included the part of LR quadrant (early apoptotic cells) and UR quadrant (late apoptotic cells). The statistical analysis is shown in the bar graphs. Data are presented as the mean ± SDM of three independent experiments. Statistically different from the control is marked with asterisk (*$p < 0.05$, **$p < 0.01$, and ***$p < 0.001$).

A B

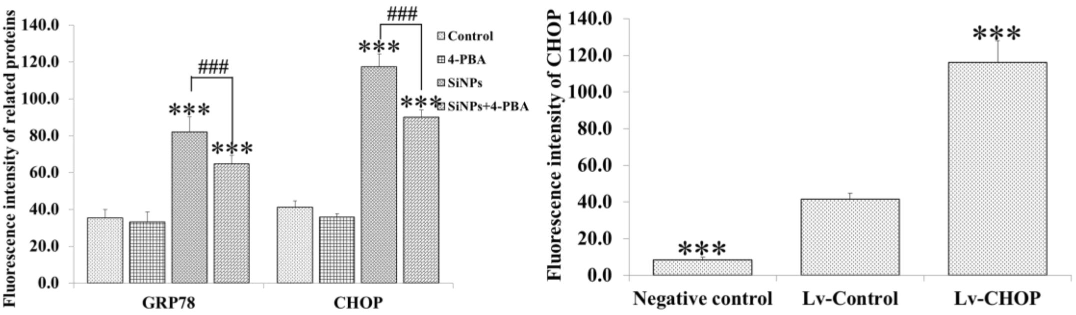

Figure S2. Analysis of fluorescence intensity in RAW 264.7 macrophage cells. (A) Analysis of fluorescence intensity of GRP78 and CHOP in RAW 264.7 macrophage cells in the Control, 4-PBA, SiNP, and SiNP + 4-PBA groups. RAW 264.7 macrophages were pretreated with 1 mM 4-PBA for 1 h, and exposed to 100 μg/mL SiNPs for 12 h. (B) Analysis of fluorescence intensity of CHOP in the normal (Negative control), Lv-CHOP, and Lv-Control groups of the stably expressed RAW 264.7 macrophage cells. The statistical analysis is shown in the bar graphs. Data are presented as the mean ± SDM of three independent experiments. Statistically different from the control or Lv-Control is marked with asterisk (***p < 0.001), respectively, and statistically different from SiNPs is marked with number sign (###p < 0.001).

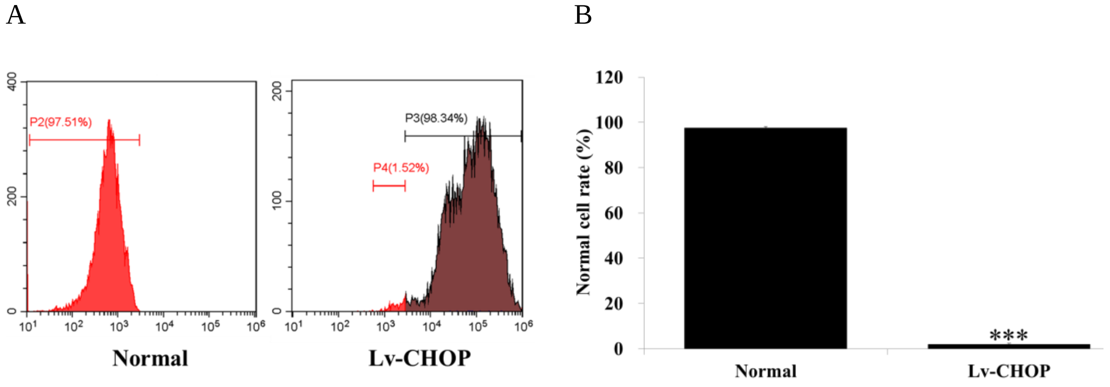

Figure S3. Detection of the transduction efficiency of CHOP lentivirus in RAW 264.7 macrophage cells via flow cytometry assay. Recombinant CHOP lentivirus was transduced into RAW 264.7 macrophage cells. The stably expressed cells of CHOP overexpression and knockdown were selected by puromycin. The transduction efficiency was detected via flow cytometry assay. The statistical analysis is shown in the bar graphs. Data are presented as the mean ± SDM of three independent experiments. Statistically different from the control is marked with asterisk (***$p < 0.001$).

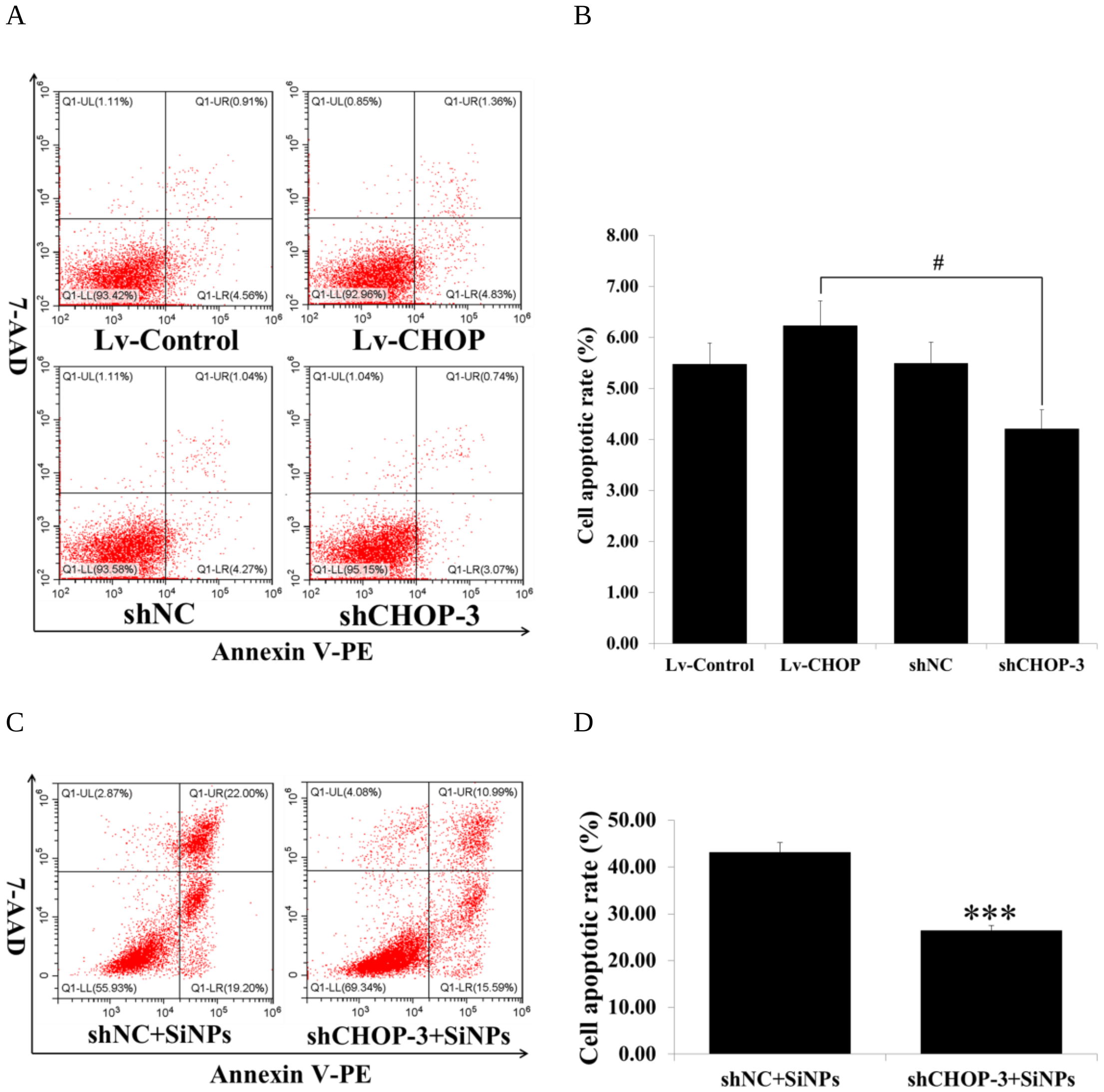

Figure S4. Detection of cell apoptosis in the stably expressed RAW 264.7 macrophage cells. (**A, B**) The stably expressed cells were no treatment. (**C, D**) The stably expressed cells were exposed to 200 μg/mL SiNPs for 24 h. Cells were stained with Annexin V-PE/7-AAD, and the apoptotic rates were determined via flow cytometry analysis. UL quadrant is the part of cell death caused by mechanical damage or necrotic cells, UR quadrant is the part of late apoptotic cells, LL quadrant is the part of the normal cells, and LR quadrant is the part of early apoptotic cells. The number of cell apoptosis included the part of LR quadrant (early apoptotic cells) and UR quadrant (late apoptotic cells). Statistical analysis of the cell death is shown in the bar graphs. Data are presented as the mean ± SDM of three independent experiments. Statistically different from the control is marked with asterisk (***$p < 0.001$), and statistically different from SiNPs is marked with number sign (#$p < 0.05$).

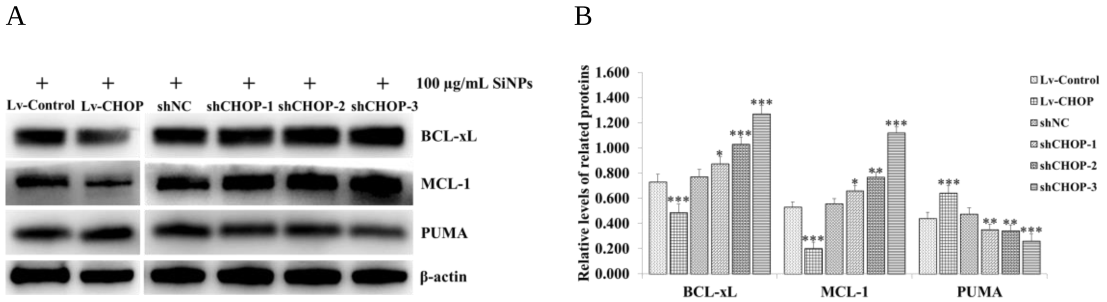

Figure S5. Effect of CHOP on the expression of the apoptosis-related proteins in RAW 264.7 macrophage cells. (**A, B**) The stably expressed RAW 264.7 macrophage cells were exposed to 100 μg/mL SiNPs for 12 h. The expression of BCL-xL, MCL-1, and PUMA were detected via western blot analysis. The analyses of the band intensity on the films are presented as the relative ratio of target proteins to β-actin, respectively. Statistical analysis is shown in the bar graphs. Data are presented as the mean ± SDM of three independent experiments. Statistically different from the control is marked with asterisk (*$p < 0.05$, **$p < 0.01$, and ***$p < 0.001$).

2. Materials and Methods

2. 1. Immunofluorescence staining of the stably expressed cells of CHOP

The stably expressed cells of CHOP were cultured on sterile cover slips placed in 24-well culture plates. The cells were then fixed in PFA solution (4%, vol/vol) for 30 min at room temperature. Following fixation, the cells were permeabilized with 0.5% TritonX-100 for 5 min and blocked with 5% BSA for 1 h and then exposed to anti-CHOP (1:250 dilutions) overnight at 4°C. After washing, the cells were incubated with anti-mouse red fluorecscent secondary antibodies (1:1000 dilutions) at 37°C for 1 h in the dark and DAPI for 10 min at room temperature. Finally, the cells were examined under a laser scanning confocal microscope (TCS SP8 STED; Wetzlar, Hessen, GER).
